# Soil bacterial diversity correlates with precipitation and soil pH in long-term maize cropping systems

**DOI:** 10.1038/s41598-020-62919-7

**Published:** 2020-04-07

**Authors:** Wenjun Tan, Junman Wang, Wenqing Bai, Jiejun Qi, Weimin Chen

**Affiliations:** Shaanxi Key Laboratory of Agricultural and Environmental Microbiology, College of Life Sciences, Northwest A&F University, Yangling, Shaanxi 712100 P. R. China

**Keywords:** Soil microbiology, Microbial ecology

## Abstract

Unraveling the key drivers of bacterial community assembly in agricultural soils is pivotal for soil nutrient management and crop productivity. Presently, the drivers of microbial community structure remain unexplored in maize cropping systems under complex and variable environmental scenarios across large spatial scales. In this study, we conducted high-throughput 16S rRNA gene sequencing and network analysis to identify the major environmental factors driving bacterial community diversity and co-occurrence patterns in 21 maize field soils across China. The results show that mean annual precipitation and soil pH are the major environmental factors that shape soil bacterial communities in maize soils. The similarities of bacterial communities significantly decreased with increasing geographic distance between different sites. The differences in spatial turnover rates across bacterial phyla indicate the distinct dispersal capabilities of bacterial groups, and some abundant phyla exhibited high dispersal capabilities. *Aeromicrobium*, *Friedmanniella*, *Saccharothrix*, *Lamia*, *Rhodococcus*, *Skermanella*, and *Pedobacter* were identified as keystone taxa. Based on the node-level and network-level topological features, members of the core microbiome were more frequently found in the center of the ecosystem network compared with other taxa. This study highlights the major environmental factors driving bacterial community assembly in agro-ecosystems and the central ecological role of the core microbiome in maintaining the web of complex bacterial interactions.

## Introduction

As an important component of terrestrial ecosystems, agricultural soils are the productive engine of the Earth and account for over one third of the global land area^1^. Microbes have an immense amount of diversity, play important roles in global biogeochemical processes, and drive the functioning of diverse ecosystems^2^. In agricultural soils, microbes are critical to crop productivity by participating in organic matter decomposition, humus formation, and nutrient transformation^3,4^. Understanding how biodiversity is generated and maintained, as well as determining the drivers of microbial distribution patterns across different scales is the central goal of microbial ecology^5^. There has been a growing number of studies on microbial biogeography in diverse environments^6–9^. Microbes are distributed worldwide; however, in a given habitat, varying environmental factors can lead to different niches and select for adapted microbes. For example, the microbial distribution can be substantially influenced by soil pH, nutrient availability, and climate conditions^10–13^.

The spatial patterns of microbial diversity can reveal the key drivers of biodiversity and help predict the risk of biodiversity loss. The distance-decay relationship (DDR) has been well documented for the spatial distributions of plant and animal communities, as the community similarity decreases with increasing geographic distance^5^. Recent studies have shown that the non-random distribution of terrestrial and aquatic microbial communities is also affected by geographic distance and exhibits distance-decay patterns across different habitats^6,7,14^. Thus, spatial distance may induce dispersal limitation, influencing the biogeography of microbes^5^. However, it is still challenging to characterize and quantify microbial communities distributed in agro-ecosystems under complex and variable environmental scenarios on a large scale. Maize (*Zea mays* L.) is one of the major cereal crops worldwide and it is widely cultivated across China, making a suitable model for assessing such broad-scale questions. In addition, maintaining a stable state of the plant community and ecosystem management would allow for observation of the influence of edaphic and other abiotic factors on soil microbial communities.

In a particular habitat type, a suite of members broadly distributed amongst microbial communities at different locations is defined as the core microbiome^7,15^. Given its ubiquity in a specific environment, discovering a core microbiome is critical to understand the assembly and stability of microbial communities^15^. Determining the factors driving assembly of the core microbiome in agricultural soils has implications for understanding the microbial functional groups in agro-ecosystems. Moreover, the interactions among microbes form a complex network in specific ecosystem niches. Microbial co-occurrence patterns can help to uncover the complex interactions of microbial communities and delineate the underlying ecological processes^16^. Network analysis can identify the keystone microbial taxa that have the greatest influence on community structure and determine the ecological roles of specific microbial groups of concern.

Here we investigated the drivers of bacterial community assembly in maize cropping systems across a large spatial scale. The main objectives of the study were: (i) to examine the bacterial community diversity and structure in maize soils by high-throughput 16S rRNA gene sequencing; ii) to identify the major environmental factors that influence bacterial community diversity, and; (iii) to identify the keystone taxa in maize cropping systems via network analysis.

## Results

### Geochemistry of maize soils

The samples included a diverse array of soil types, including sandy clay loam, clay loam, loamy clay, sandy loam, silty clay loam, and loam (Table 1). The soils were slightly acidic to alkaline, with the pH varying from 5.23 to 8.86 (pH > 7 in 15 of 21 samples). There was a wide variation in the mean annual precipitation across different sites (61.3–2,162.8 mm). The pairwise distances between the sites ranged from 87 to 4,130 km (Table S1, Supporting Information). There were no correlations between edaphic factors and geographic distances (*P* > 0.1; Mantel test). However, the two climatic factors (mean annual temperature and precipitation) were negatively correlated with latitude (*P* < 0.05; Spearman correlation).

**Table 1:** Environmental parameters of the 21 soil samples collected from maize cropping systems across China.

Site	Edaphic						Geographic		Climatic		Soil type
	pH	AK^a^ (mg/kg)	OC (mg/kg)	TN (mg/kg)	AN (mg/kg)	AP (mg/kg)	Latitude (°)	Longitude (°)	PRE (mm)	TEM (°C)	
1	7.68	117.10	18.26	0.78	43.75	61.98	18.65	109.67	2162.8	24.8	sandy clay loam
2	5.46	167.70	26.40	1.06	103.25	15.83	25.00	101.51	937.2	16.2	clay loam
3	7.93	125.20	14.87	0.68	35.00	17.44	27.92	112.75	1377.0	17.4	loamy clay
4	8.00	116.10	10.42	0.44	19.25	8.30	29.23	120.05	1386.6	17.7	sandy loam
5	7.96	145.40	29.72	0.96	98.00	30.48	29.52	106.66	1108.0	18.4	sandy loam
6	8.39	55.10	3.16	0.44	3.50	3.39	31.88	106.16	1013.5	16.6	loamy clay
7	5.23	135.30	13.62	0.56	94.50	21.82	33.28	114.30	863.1	14.8	clay loam
8	8.33	198.20	16.49	0.84	31.50	14.17	34.36	109.12	569.4	13.8	clay loam
9	8.65	234.70	16.75	0.50	35.00	17.03	35.15	111.23	519.2	13.2	silty clay loam
10	7.87	86.50	14.86	0.56	87.50	9.45	35.95	116.57	614.3	14.0	clay loam
11	8.72	208.30	11.67	0.44	49.00	4.92	36.25	106.39	425.5	6.9	clay loam
12	8.74	102.80	5.86	0.16	5.25	4.45	36.59	107.27	409.5	9.2	silty clay loam
13	6.37	288.20	15.25	0.62	31.50	87.36	36.64	119.41	546.5	12.8	loamy clay
14	5.83	135.40	37.52	1.24	63.00	175.37	37.36	114.79	478.2	13.2	sandy loam
15	8.76	207.30	13.73	0.50	5.25	38.06	38.45	77.25	61.3	12.0	sandy loam
16	8.50	80.70	23.17	0.68	29.75	127.64	39.52	112.53	407.3	6.1	sandy loam
17	8.86	248.90	18.94	0.78	87.50	52.70	39.88	117.73	623.8	11.9	clay loam
18	7.04	105.00	10.06	0.56	21.00	14.10	39.94	121.52	617.5	9.9	sandy clay loam
19	8.45	143.40	8.81	0.40	175.00	57.88	40.75	111.97	396.5	7.3	loamy clay
20	8.04	323.00	34.16	1.00	47.25	56.16	43.87	126.75	580.0	5.0	loamy clay
21	8.18	144.40	26.59	1.00	66.50	28.88	46.51	124.96	434.6	3.8	sandy loam

^a^AK, available potassium; OC, organic carbon; TN, total nitrogen; AN, available nitrogen; AP, available phosphorus; PRE, mean annual precipitation; TEM, mean annual temperature.

### Distribution of bacterial taxa

**Figure 1:**
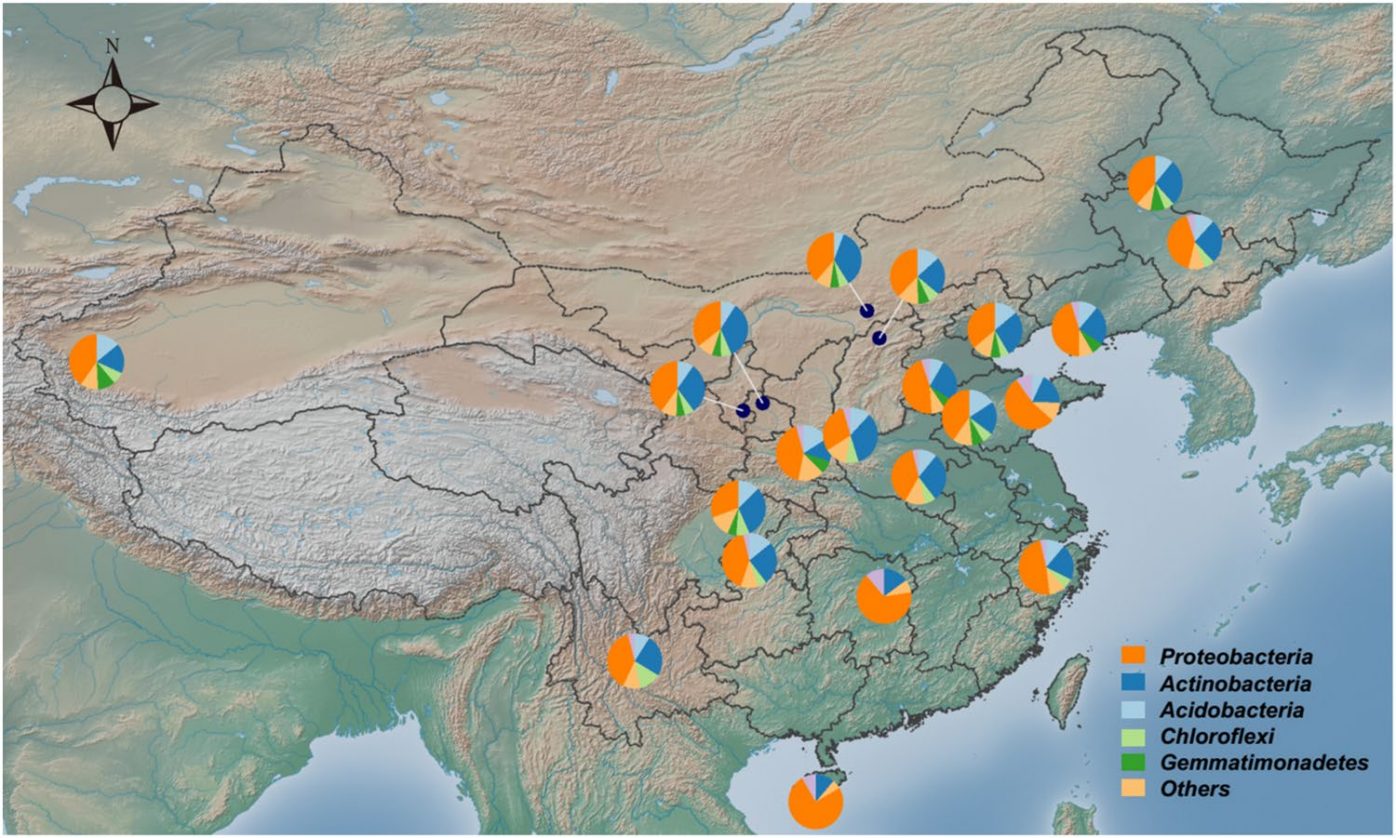
Locations of the 21 sampling sites with their phylum-level distributions of soil bacterial communities in maize cropping systems across China. The map was generated using GenGIS software (Version II, http://kiwi.cs.dal.ca/GenGIS).

We obtained 613,504 high-quality sequences in total, with 18,844 to 36,891 sequences per sample (mean = 29,214). There were 5,318 operational taxonomic units (OTUs), 5,307 of which were affiliated with the bacterial domain, and the remaining 11 with the archaeal domain. Five dominant bacterial phyla (relative abundance >5%) accounted for 89.7% of the total sequences: Proteobacteria (43.1%), Actinobacteria (24.4%), Acidobacteria (10.7%), Chloroflexi (6.0%), and Gemmatimonadetes (5.5%). At the class level, Proteobacteria was dominated by Betaproteobacteria, Alphaproteobacteria, Gammaproteobacteria, and Deltaproteobacteria; Actinobacteria was dominated by Actinobacteria and Thermoleophilia (overall relative abundance, 21.88%). The dominant archaea was *Candidatus Nitrososphaera* (Crenarchaeota phylum), which accounted for 0.11% of all sequences. Additionally, Bacteroidetes, Planctomycetes, Cyanobacteria, Firmicutes, Nitrospirae, Armatimonadetes, and Verrucomicrobia were detected in most samples at low relative abundances (<5%) (Fig. 1).

**Figure 2:**
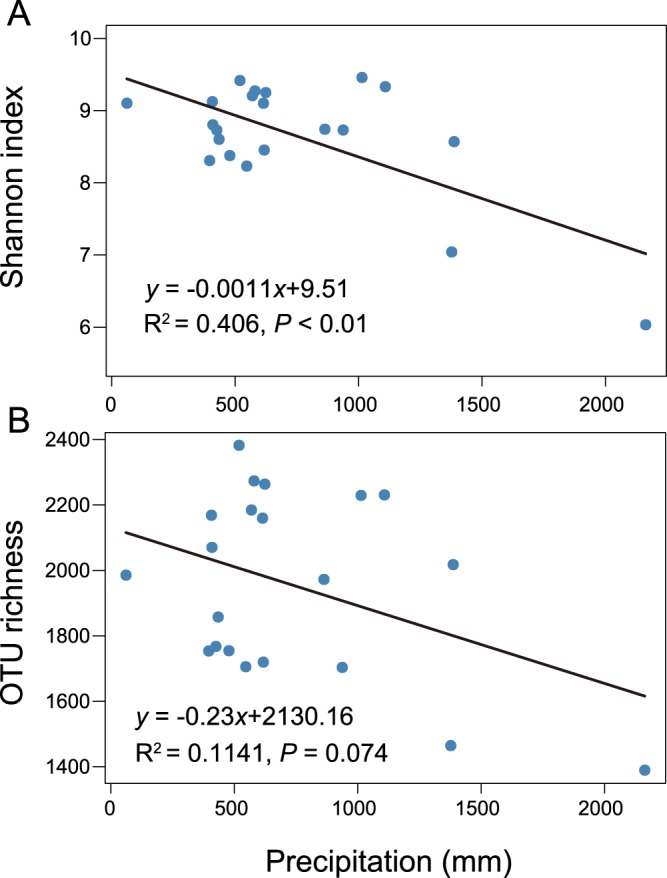
The linear regression relationship between mean annual precipitation and either Shannon-Wiener index (**A**) or operational taxonomic unit (OTU) richness (**B**) of soil bacterial communities in maize cropping systems (n = 21).

**Figure 3:**
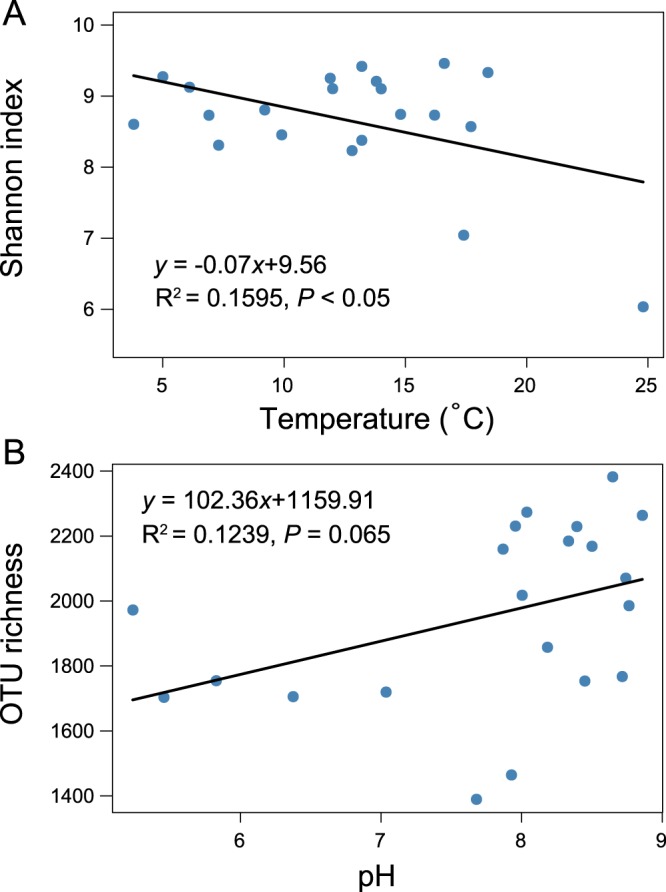
The linear regression relationships between: mean annual temperature and Shannon–Wiener index of soil bacterial communities (**A**), and; soil pH and operational taxonomic unit (OTU) richness of soil bacterial communities (**B**) in maize cropping systems (n = 21).

Alpha diversity, measured by OTU richness (r = −0.398, *P* < 0.1, marginally significant) and Shannon–Wiener index (r = −0.66, *P* < 0.01, significant), was negatively correlated with mean annual precipitation (Table S2, Supporting Information). The Shannon–Wiener index was also negatively correlated with mean annual temperature (r = −0.449, *P* < 0.05, significant). Meanwhile, there was a marginally significant positive correlation between OTU richness and soil pH (r = 0.409, *P* < 0.1, marginally significant). These significant correlations were confirmed by the linear regression relationships, as shown in Figs. 2 and 3.

### Environmental factors influencing bacterial community structure

**Figure 4:**
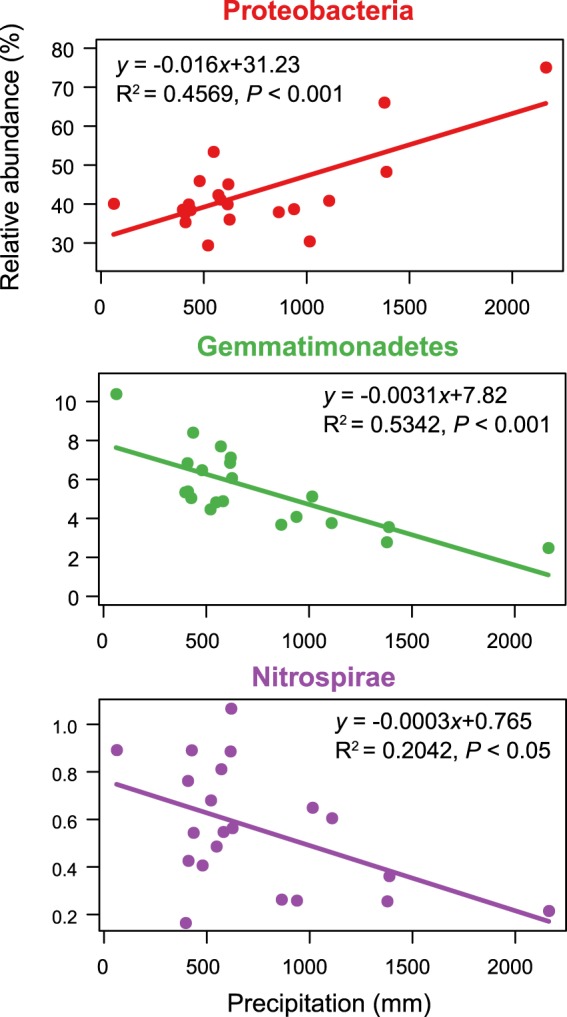
The linear regression relationships between the relative abundances of the dominant bacterial phyla and mean annual precipitation in maize cropping systems (n = 21).

Major environmental factors that shaped the microbial communities were determined by Constrained analysis of principal coordinates (CAP) based on Weighted Unifrac distance (Fig. S1, Supporting Information**)**. Mean annual precipitation and soil pH were significantly linked with bacterial community structure (PERMANOVA; Supplementary Table S3). Additionally, mean annual precipitation, which explained higher degree of variance (R^2^ = 0.277), was a major driver of bacterial community structure. The effect of mean annual precipitation on the bacterial community structure was evident at a low taxonomic resolution, as the relative abundances of dominant phyla were significantly changed along with the annual precipitation gradient, as determined by linear regression relationships (Fig. 4). With the increasing of mean annual precipitation, the relative abundance of Proteobacteria was significantly increased and the relative abundance of Gemmatimonadetes, and Nitrospirae significantly decreased.

**Figure 5:**
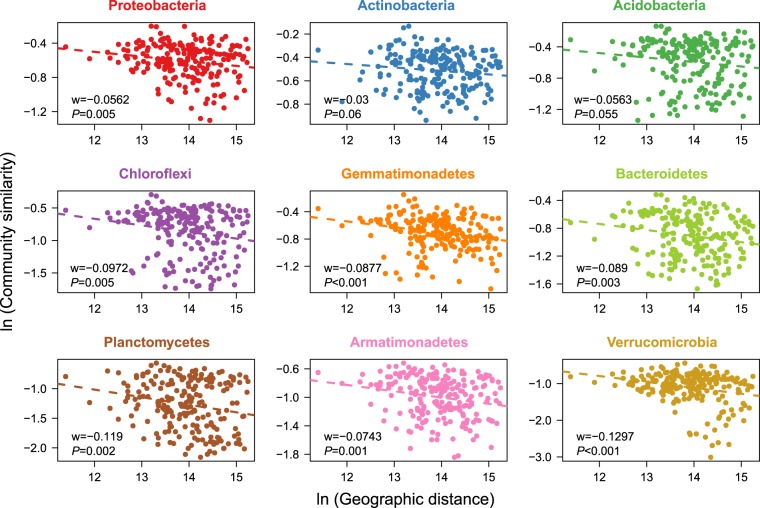
The distance-decay relationships of dominant bacterial phyla in maize cropping systems (n = 21 × 21). A “w” represents the slopes of the linear regression curve, and a “*P*” represents the significance of the linear regression model.

The influence of spatial distance on the variation in community structure was estimated by DDR, a distance-decay similarity model. Significant negative DDRs were found in a linear regression using beta diversity (Weighted Unifrac distance; Fig. S2, Supporting Information). At different taxonomic levels, many dominant phyla also exhibited significant DDRs. Interestingly, Planctomycetes and Verrucomicrobia showed steeper slopes than other phyla (Fig. 5).

Given the important influence of soil types on the bacterial community structure based on prior knowledge, we estimated the effect of soil texture on bacterial community structure. Soil samples were divided into three groups, according to the different soil types, including clay loam, loamy clay and sandy loam. Based on PCoA analysis, we did not observe clear clustering of these samples, according to soil types (Fig. S3, Supporting Information). This was confirmed by ANOSIM and permutational multivariate analysis of variance (ADONIS) tests confirmed that no significant difference was observed in bacterial community structure among soil types (*P* > 0.1), Furthermore, bacterial alpha diversity showed no significant difference among soil types (*P* > 0.1), estimated via one-way analysis of variance. These results indicated that soil types did not influence the diversity and structure of bacterial community in Maize soils of present work.

### Bacterial community assembly of the core microbiome

We identified 421 core taxa in seven phyla and seventy genera. In general, *Janthinobacterium*, *Ramlibacter*, *Kaistobacter*, *Lysobacter*, and *Thermomonas* were the predominant members of the core microbiome from the Proteobacteria phylum. *Nocardioides, Streptomyces*, and *Rhodococcus* were the predominant members of the core microbiome from the Actinobacteria phylum (Fig. S4, Supporting Information). Based on Bray–Curtis dissimilarity, the core microbiome contributed to a substantial fraction (mean = 64.45%, range = 39.32–81.83%) of community dissimilarity despite accounting for only 7.92% of the total OTUs (Fig. S5, Supporting Information).

**Figure 6:**
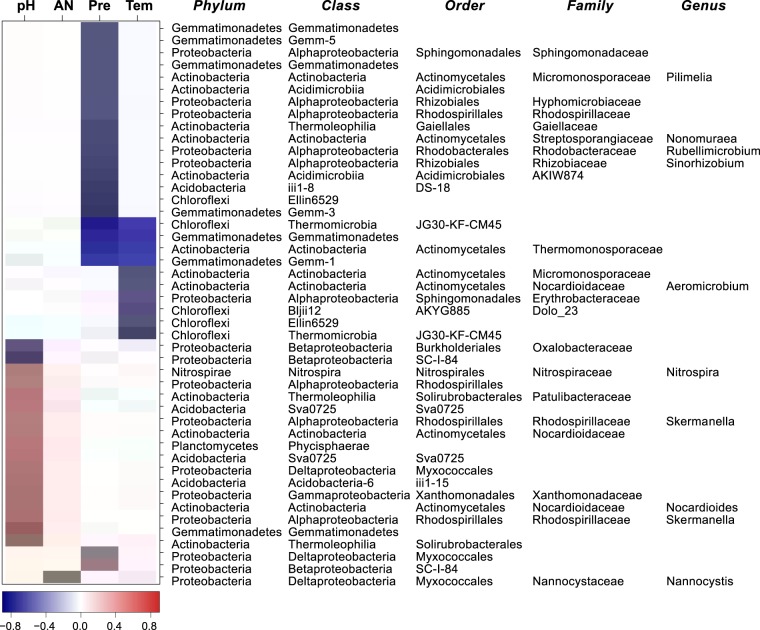
Spearman correlations between the core microbiome and environmental factors in maize cropping systems. The values of correlation coefficients are indicated according to the scale bar. Only significant (*P* < 0.05) and strong (correlation coefficient, r > 0.6 or <–0.6) correlations are shown; others are left blank. AN, available nitrogen; Pre, Mean annual precipitation, and; Tem, Mean annual temperature.

To explore the influences of environmental factors on the assembly of the core microbiome, we estimated the associations between the core microbiome and environmental factors by correlation analysis. There were significant [false discovery rate (FDR)-corrected *P* < 0.05] and strong (r > 0.6 or <–0.6) correlations with four environmental factors (Fig. 6). For example, *Nitrospira* and *Skermanella* were positively correlated with soil pH; *Nannocys* was positively correlated with available nitrogen, and; *Pilimelia*, *Nonomuraea*, *Rubellimicrobium*, and *Sinorhizobium* were negatively correlated with mean annual precipitation; *Aeromicrobium* was negatively correlated with mean annual temperature.

### Bacterial co-occurrence patterns and keystone taxa

First, we structured a soil bacterial network based on correlations between OTUs to explore the overall distribution of co-occurrence patterns and the ecological role of the core microbiome. The resulting network consisted of 5,312 nodes (i.e., OTUs) and 181,157 edges (mean = 34 edges per node). Topological analysis showed that the APL was 3.237 edges with a ND of 4.401 edges, and the CC was 0.162 (Table S4, Supporting Information). The structural properties of the real-world network were greater than those of an identically sized random Erdös–Réyni network (APL = 2.398, CC = 0.0128, and ND = 3). These suggest that the real bacterial network was non-randomly distributed and had a highly connected topological structure.

**Figure 7:**
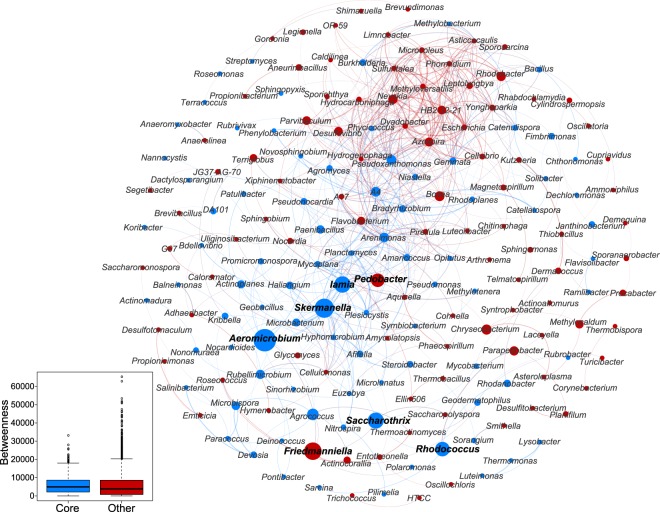
Network of co-occurring bacterial genera in maize cropping systems based on correlation analysis. A connection stands for a strong (Spearman r > 0.6) and significant (*P* < 0.01) correlation. The size of each node is proportional to the betweenness centrality value; the thickness of the connection between two nodes (edge) is proportional to the value of the Spearman correlation coefficient. The nodes colors are based on categorization as a core (“Blue”) or other (“red”) taxa. The boxplot shows the comparison of betweenness centrality values between the core and other taxa in maize cropping systems. The values were significantly different between the two groups based on a Wilcoxon Rank Sum test (*P* < 0.05).

The betweenness centrality values were significantly higher in the core microbiome nodes than in other nodes (Fig. 7). This suggests that core taxa were more likely to be located in central position within the network. To verify this observation, a sub-network was generated for the core microbiome and network-level topological features were analyzed (Table 2). The values of topological features were higher in the core microbiome sub-network than in the other sub-network, indicating that the former sub-network was more connected and complex than the latter sub-network.

**Table 2:** Network-level topological features of the sub-networks for the core and other taxa in maize cropping systems.

	Clustering coefficient	Average path length	Network diameter	Graph density
Core	0.462	3.473	0.055	6.506
Others	0.126	3.247	0.013	4.410

The co-occurrence patterns of soil microbes were examined at the genus level and keystone genera were identified. The genus-level network consisted of 184 nodes and 639 edges (Fig. 7). The nodes primarily belonged to four bacterial phyla: *Proteobacteria*, *Actinobacteria, Firmicutes*, and *Bacteroidetes*. Seven genera were considered keystone based on betweenness centrality scores. *Aeromicrobium*, *Friedmanniella*, *Saccharothrix*, *Lamia*, and *Rhodococcus* were keystone genera from the Actinobacteria phylum; *Skermanella* is from the Proteobacteria phylum, and *Pedobacter* was a keystone genus from the Bacteroidetes phylum. Five of the keystone genera were members of the core microbiome (Fig. S6, Supporting Information).

## Discussion

Given the global role of microbes in the environment, determining the drivers of microbial community assembly is an important issue in microbial ecology^17^. Microbes inhabiting agricultural soils are closely linked to crop productivity through complex biogeochemical processes^18^. In this study, we investigated the major environmental factors that drive bacterial community assembly in maize soils across a large spatial scale with a latitude gradient.

Maize, which possesses exceptional phenotypic and molecular diversity, can grow in diverse soils with a pH range of 5.7–8.0^19^. In the present study, the maize soils were slightly acidic to alkaline, within the suitable pH range for growth of maize. The variation of soil pH might be related with the wide variety of soil textures. In addition, most of our soil samples (71%) were circumneutral to alkaline (pH > 7). Bacterial communities can be shaped by complex environmental factors. Significant correlations have been frequently found between the pH and bacterial alpha-diversity in soils^20,21^ and lake sediments^22^, indicating that pH is a predictor for soil microbial diversity. In the present study, OTU richness showed a marginally significant and positive correlation with soil pH in the range of 5.23–8.86, in agreement with previous observation in British soils over a broader pH range from 3 to 9^20^. Soil pH could affect the bioavailability of carbon and nitrogen substrate as well as toxic metals, indirectly influencing soil microbes^23^. The richness and diversity of bacterial communities are key maintainers for the productivity and stability of agricultural soil ecosystems^3^.

Two climatic factors, mean annual precipitation and temperature, also predicted bacterial diversity in the maize soils. These two variables were negatively correlated with the Shannon–Wiener index of soil bacterial communities in the current study, although we also acknowledged that the significant relationship between the Shannon index and the average annual precipitation may be driven by two outliers. This result could be supported by a continental-scale study across eastern China^24^, which reported higher bacterial diversity at high latitudes in maize fields. However, conflicting results were reported in natural terrestrial ecosystems that soil bacterial diversity was positively or not correlated with temperature^10,13^. The discrepancy may be associated with the complex and heterogeneous environments across different habitats across multiple scales.

In the present study, both mean annual precipitation and soil pH were significantly linked to bacterial community structure in maize soils based on beta-diversity metrics. Similarly, annual precipitation and temperature were found significantly linked to the functional structure of microbial communities in a soil transplant experiment designed to simulate climate change^25^. Annual precipitation was a larger contributor to variation in bacterial community structure than soil pH in the maize cropping systems. This might be attributed to the large variability of precipitation among the sampling sites, which were across a large spatial scale and covered large landmass and various climate regions in China. Precipitation could change soil moisture, and sudden change in moisture is stressful to microbes, as they must expend energy to regulate osmotic pressure to their microenvironment^26^. Additionally, annual precipitation could compensate for water tables and affect redox condition seasonality^27^. In addition, climate factors can change soil geochemistry and thereby impact microbial community structure. For example, a national soil survey of Scotland revealed that soil pH and precipitation altered the diversity of ammonia oxidizer communities and their capacity for nitrification^11^. Moreover, the combination of warming and decreased precipitation altered bacterial community structure in a Tibetan plateau alpine grassland after a 1-year manipulation^28^.

The above findings suggest that precipitation is a major factor impacting bacterial community assembly in soils; this is supported by our observations in the current study. A few dominant phyla were significantly correlated with mean annual precipitation in the maize cropping systems. With increasing annual precipitation, Proteobacteria increased, while Gemmatimonadetes, and Nitrospirae decreased in maize soils. Similarly, altered precipitation was found to specifically impact Gammaproteobacteria in alpine grassland^28^. Another study found that Gemmatimonadetes was inversely correlated with soil moisture^29^.

An important issue in biogeography is estimating whether spatial distance creates genetic variation^5^. The DDR estimates the variation in beta-diversity across spatial scales^30,31^. In the present study, the variation in soil bacterial community decreased linearly with increasing geographic distance, indicating the spatial structure of bacterial communities in maize soils. This result could be supported by other studies demonstrating the robust DDRs of microbial communities in agricultural fields^32,33^.

Various spatial scales could result in a difference in the distance-decay slope between habitats due to the dependence of distance-decay on the spatial scale^34,35^, which is known to affect the assembly of soil bacterial communities in wheat fields^33^. Here we found that the distance-decay slope (w = −0.043) in maize soils with an interval of 4129.6 km was steeper than in dryland habitats of northern China across a 4000 km transect^35^, including alpine grassland (w = −0.041), desert (w = −0.017), desert grassland (w = −0.014), and typical grassland (w = −0.017). The slopes of these relationships across habitats can differ, reflecting varying rates of species turnover in their habitats^35^. In particular, agricultural fields are typical human-managed terrestrial ecosystems, resulting in distinct microbial community assembly patterns compared to natural ecosystems^36^. Agricultural soils can form unique, ephemeral habitats across local sites during long-term tillage^37^, thus generating more spatially structured microbial communities across large scales. Dispersal limitations, which can be attributed to geographic distances, reflect a stochastic process that influences the beta diversity and biogeographic patterns of microbes^5^. This is in accordance with previous findings in diverse habitats that microbial distributions are not only driven by deterministic process (i.e., environmental heterogeneity), but can also be governed by stochastic process (i.e., dispersal limitations)^6,7^.

Furthermore, a number of bacterial phyla showed significant DDRs with differing slopes in the maize soils, indicating their distinct turnover rates along the spatial gradient. The dominant phyla Proteobacteria, Actinobacteria, and Acidobacteria had lower slopes and stronger dispersal capabilities, while the less abundant phyla Planctomycetes and Verrucomicrobia showed lower dispersal capabilities. Dispersal capabilities may be linked to phylum abundance. Abundant bacteria are readily dispersed, as many individual cells can potentially be involved in a dispersal event; rare bacteria with lower abundances should show lower dispersal rates than abundant taxa^38^. Verreydt, *et al*.^39^ found that the dispersal capabilities of bacterial groups varied greatly in aquatic habitats, similar to our observations in maize soils.

In particular, we found that Proteobacteria and Gemmatimonadetes were correlated to both geographic distance and environmental factors, although no autocorrelations were found between geographic distance and edaphic factors. Thus, the change in the relative abundances of particular microbes may be ascribed to both dispersal limitations and environmental heterogeneity. Stochastic processes generate random variation in the relative abundances of species (i.e., dispersal limitation and ecological drift) and create patchiness in community composition^40^. Deterministic processes indicate that environmental selection including abiotic and biotic factors could determine the community assembly^41^. The above results indicate the combined influence of deterministic and stochastic processes on the bacterial community assembly in maize cropping systems.

Network analysis was used to explore the bacterial co-occurrence patterns and provide insights into bacterial interactions in soils from maize cropping systems. When compared with a random network, the non-random co-occurrence of the bacterial communities was observed across the maize soils; this indicates the role of deterministic processes in bacterial community assembly of agro-ecosystems^7^. In this study, the core microbiome was broadly distributed and accounted for a high fraction of community beta diversity but a low proportion of total OTUs in the maize soils. According to the node-level (betweenness centrality) and network-level topological features, we found that members of the core microbiome were more often located in central ecological positions than other taxa. A more connected and complex sub-network was therefore generated for the core microbiome. Betweenness centrality scores can be used to determine the most influential taxa (e.g., keystone species) that are responsible for maintaining connectivity within the ecological network^42^. Thus, the core microbiome might play a vital role in maintaining the complex connections between microbes in agro-ecosystems.

In a co-occurrence network analysis, the topological features of nodes are used to determine the potential importance of microorganisms, such as keystone species^43–45^. Different with core taxa, keystone species were determined via network analysis. Based on betweenness centrality scores, *Aeromicrobium*, *Friedmanniella*, *Saccharothrix*, *Lamia*, *Rhodococcus*, *Skermanella*, and *Pedobacter* were considered keystone taxa in the maize soils. *Aeromicrobium* and *Rhodococcus* are characteristic of herbicide metabolism^46^. *Friedmanniella* has been detected in lignocellulose degradation of composted agricultural waste^47^. *Saccharothrix* possesses antifungal activity^48^. *Pedobacter* has been isolated from a herbicide-contaminated soil^49^. These keystone taxa may play versatile roles in soil ecological processes in agro-ecosystems.

Sources of uncertainties should be noted when interpreting the results of this study. Firstly, number of samples is small and there are no duplicates at each site, which may affect our results. In the further work, three composite samples per site would be taken into account. Second, although we sampled at the same time, we failed to consider the specific management system of each site, such as the variety of corn, the amount of fertilizer applied and the farming method. Therefore, in addition to natural rainfall, the source of water is then agricultural irrigation, but our study did not include artificial water supply due to large-scale sampling, which may still influence the results.

In conclusion, this is a detailed study of soil bacterial communities in maize cropping systems across a continental scale in China. We determined the major environmental factors correlating with bacterial community diversity and explored the bacterial co-occurrence patterns in 21 maize soils across China. Mean annual precipitation and soil pH were the main factors that shaped the bacterial communities. Different spatial turnover rates of microbes suggest distinct dispersal capabilities of different bacterial groups. The central ecological role of the core microbiome suggests its vital roles in promoting soil ecological processes and maintaining the complex bacterial network. The non-random co-occurrence patterns and identification of keystone taxa provide new insights into microbial community assembly in agro-ecosystems. Furthermore, microbial taxonomic and functional data across temporal scales should be integrated to better characterize the patterns of microbial biogeography and dynamics in agro-ecosystems. Particularly, the investigation the specific distribution of the core microbiomes across different crops agro-ecosystems might improve our understanding of nutrient management and crop health in agro-ecosystems, which should be further explored.

## Methods

**Soil sampling and data collection**. Twenty-one soil samples were collected from maize fields over 10 years old in 19 provinces across China (18.62°–46.51° N; 77.25°–126.75° E) during July–August 2014 (Fig. 1**)**. Since the maize growing period varied across regions, we collected the bulk soils rather than rhizosphere soils. At each sampling site, five random soil cores were taken from a depth of 0–15 cm in a 100 × 100 m plot and combined to form a composite sample. The samples were immediately transported to the laboratory on dry ice, where they were sieved through a 2.0-mm mesh to remove plant debris and rocks^36^. Subsamples for physicochemical analysis were air-dried. Soil pH, organic carbon, total nitrogen, available nitrogen, available phosphorus, available potassium, and texture were analyzed using standard soil testing procedures^50^. Mean annual air temperature and precipitation data were obtained from the WorldClim database (http://www.worldclim.org). Subsamples for microbial analysis were stored at −80 °C until DNA was extracted.

### DNA extraction and Illumina sequencing analysis of 16S rRNA gene amplicons

Total genomic DNA was extracted from 0.5 g of each soil sample using the MP FastDNA^®^ SPIN Kit for soil (MP Biochemicals, Solon, OH, USA) following the manufacturer’s instructions. The V4 and V5 hypervariable regions of the bacterial 16S rRNA gene were PCR amplified using the primer pair 515F and 907R^7^. The purified PCR amplicons were sequenced using an Illumina Miseq (300-bp paired-end reads) platform (Illumina Inc., San Diego, CA, USA). The acquired sequences were filtered by quality according to Caporaso, *et al*.^51^. Chimeric sequences were removed using the UCHIME algorithm^52^ in the USEARCH package v. 6.1544^53^. Sequences were grouped by taxonomy and assigned to OTUs at the 97% sequence identity level using the UPARSE package (http://drive5.com/uparse/)^54^. After singleton removal, representative sequences were taxonomically assigned using the Ribosomal Database Project naïve Bayesian rRNA classifier within the SILVA database (release 128) at an 80% confidence threshold^51^. All sequences were deposited in the NCBI Sequence Read Archive database (Biosample number: SAMN06105854-SAMN06105874).

### Data analyses

Alpha diversity (OTU richness and Shannon–Wiener index) and beta diversity were calculated using QIIME (http://qiime.org/index.html) based on a subsample containing a minimum number of sequences (18,844)^7^. The same subsample was used to calculate the pairwise Weighted UniFrac distances between samples, i.e., bacterial community structure. A soil sampling map showing the phylum-level distribution of bacterial communities in pie charts was generated using GenGIS II^55^.

To identify the major environmental attributes that shape soil bacterial communities, Constrained analysis of principal coordinates (CAP) and permutational MANOVA were performed between environmental factors and bacterial community structure (i.e., Weighted Unifrac distance), using capscale and anova procedure in the “vegan” package^56^. To test the influence of spatial distance on the variation in community structure, we used DDR to explore the correlations between community similarity and geographic distance^35^. The DDR was estimated for each pairwise set of samples as the slope of the linear least-squares regression curve between geographic distance and bacterial community similarity (based on 1 – dissimilarity of the Weighted UniFrac distance matrix). In addition, the DDRs were calculated for different bacterial phyla to estimate their turnover rates along the spatial gradient.

The partitioned Bray-Curtis dissimilarity between two samples was calculated as the fraction of beta diversity attributed to the core microbiome (defined as bacterial taxa present in all soil samples, n = 21). The core taxa were used to calculate the summation in the numerator of the Bray-Curtis dissimilarity expression, while all of the taxa were used to calculate the scaling summation in the denominator^57,58^. Spearman correlations between the core microbiome and environmental factors were estimated to explore the influences of environmental factors on the assembly of the core microbiome, which were displayed by heatmap.

Network analysis was carried out to explore the co-occurrence patterns of bacterial taxa and the ecological role of the core microbiome^32,59^. Spearman correlations between every two OTUs were estimated, and statistically robust correlations with the correlation coefficient (r) > 0.6 and false discovery rate (FDR)-corrected *P*-value < 0.01 were selected to form a correlation network. Each node in the network represents an OTU and each edge stands for a statistically robust correlation between OTUs. Average path length (APL), network diameter (ND), graph density (GD), and clustering coefficient (CC) were calculated to describe network topology using the IGRAPH package^60^ in R environment. Meanwhile, 10,000 random Erdös–Réyni networks were generated with random probability of connections assigned to any node and compared with the real-world network in terms of topology. Furthermore, sub-network was generated for the core microbiome using the subgraph procedure in the IGRAPH package^60^, and network-level topology was analyzed to verify the central position of the core microbiome in the network.

In our study, we have applied the most common normalizations, namely total sum scaling (i.e., converting counts to proportions by dividing each species count in a sample by the total sum of counts from within that sample) and subsampling^61^, both of which effectively convert counts into relative measures of abundance^62^. We acknowledge that the normalization is important when calculating correlations among OTUs, as mentioned in Carr, *et al*.^62^. In addition, the state-of-the-art methods such as SparCC, SPIEC-EASI and FlashWeave seem to be the most appropriate options to construct the network. In the future work, these new methods and normalization of microbial taxa should be taken into account when conducting the network analysis.

To further explore the co-occurrence patterns of soil microbes at the genus level and identify the keystone genera, the co-occurrence network at the genus level was constructed. The nodes represent genera and edges stand for strong (r > 0.6) and significant (FDR-corrected *P* < 0.01) Spearman correlations between genera. The networks were visualized using Gephi. The genera with the highest betweenness centrality (a measure for the relevance of a node as capable of holding together communicating nodes) were considered keystone species^42^.

Unless otherwise indicated, all statistics analyses were performed in the R environment (http://www.r-project.org).

## Supplementary information

Supplementary information.

## Data Availability

All sequences were deposited in the NCBI Sequence Read Archive database (Biosample number: SAMN06105854-SAMN06105874).
