# Supplementary material for: Soil bacterial diversity correlates with precipitation and soil pH in long-term maize cropping systems
Source: Sci Rep. 2020 Apr 7;10:6012. doi: 10.1038/s41598-020-62919-7 (PMC7138807; doi:10.1038/s41598-020-62919-7)
Supplement: Supplementary file 1 — Supplementary information. [file 41598_2020_62919_MOESM1_ESM.docx]

**Supplementary materials for**

**Soil bacterial diversity correlates with precipitation and soil pH in long-term maize cropping systems**

**Running title:** **Soil bacterial diversity in maize fields**

**Wenjun Tan^1^, Junman Wang^1^, Wenqing Bai, Jiejun Qi & Weimin Chen**

Shaanxi Key Laboratory of Agricultural and Environmental Microbiology, College of Life Sciences, Northwest A&F University, Yangling, Shaanxi 712100, P. R. China. Correspondence and request for materials should be addressed to Weimin Chen (Tel: +86 29 87092262; Fax: +86 29 87092262; email: chenwm029@nwsuaf.edu.cn)

^1^ Wenjun Tan and Junman Wang contributed equally

**Table S1** A pairwise geographic distance (km) matrix of the 21 soil samples used in the study.

| **Site** | 1 | 2 | 3 | 4 | 5 | 6 | 7 | 8 | 9 | 10 | 11 | 12 | 13 | 14 | 15 | 16 | 17 | 18 | 19 | 20 |
| --- | --- | --- | --- | --- | --- | --- | --- | --- | --- | --- | --- | --- | --- | --- | --- | --- | --- | --- | --- | --- |
| 2 | 1100 |  |  |  |  |  |  |  |  |  |  |  |  |  |  |  |  |  |  |  |
| 3 | 1079 | 1166 |  |  |  |  |  |  |  |  |  |  |  |  |  |  |  |  |  |  |
| 4 | 1580 | 1894 | 728 |  |  |  |  |  |  |  |  |  |  |  |  |  |  |  |  |  |
| 5 | 1248 | 716 | 621 | 1299 |  |  |  |  |  |  |  |  |  |  |  |  |  |  |  |  |
| 6 | 1514 | 891 | 774 | 1363 | 267 |  |  |  |  |  |  |  |  |  |  |  |  |  |  |  |
| 7 | 1693 | 1546 | 615 | 709 | 838 | 779 |  |  |  |  |  |  |  |  |  |  |  |  |  |  |
| 8 | 1750 | 1275 | 796 | 1180 | 587 | 390 | 494 |  |  |  |  |  |  |  |  |  |  |  |  |  |
| 9 | 1843 | 1466 | 818 | 1060 | 760 | 595 | 351 | 212 |  |  |  |  |  |  |  |  |  |  |  |  |
| 10 | 2042 | 1886 | 964 | 816 | 1171 | 1062 | 363 | 701 | 492 |  |  |  |  |  |  |  |  |  |  |  |
| 11 | 1985 | 1336 | 1104 | 1497 | 750 | 487 | 795 | 325 | 454 | 916 |  |  |  |  |  |  |  |  |  |  |
| 12 | 2011 | 1402 | 1094 | 1446 | 789 | 534 | 739 | 299 | 391 | 837 | 87 |  |  |  |  |  |  |  |  |  |
| 13 | 2218 | 2140 | 1155 | 827 | 1427 | 1328 | 598 | 966 | 756 | 266 | 1166 | 1084 |  |  |  |  |  |  |  |  |
| 14 | 2142 | 1865 | 1068 | 1028 | 1153 | 998 | 456 | 611 | 403 | 223 | 759 | 674 | 418 |  |  |  |  |  |  |  |
| 15 | 3828 | 2728 | 3483 | 4050 | 2876 | 2718 | 3368 | 2877 | 3034 | 3471 | 2580 | 2647 | 3694 | 3277 |  |  |  |  |  |  |
| 16 | 2339 | 1917 | 1291 | 1336 | 1236 | 1026 | 712 | 649 | 500 | 533 | 651 | 565 | 683 | 311 | 3035 |  |  |  |  |  |
| 17 | 2487 | 2243 | 1408 | 1204 | 1533 | 1370 | 796 | 980 | 778 | 449 | 1072 | 985 | 389 | 380 | 3467 | 447 |  |  |  |  |
| 18 | 2629 | 2499 | 1562 | 1200 | 1783 | 1646 | 982 | 1262 | 1052 | 621 | 1386 | 1299 | 411 | 652 | 3783 | 771 | 324 |  |  |  |
| 19 | 2470 | 2004 | 1430 | 1477 | 1340 | 1116 | 857 | 754 | 627 | 668 | 698 | 617 | 791 | 449 | 2970 | 145 | 498 | 815 |  |  |
| 20 | 3229 | 3104 | 2172 | 1735 | 2390 | 2238 | 1598 | 1848 | 1644 | 1236 | 1924 | 1837 | 1017 | 1242 | 4130 | 1275 | 869 | 615 | 1263 |  |
| 21 | 3403 | 3172 | 2329 | 1970 | 2468 | 2287 | 1728 | 1898 | 1709 | 1368 | 1918 | 1835 | 1191 | 1319 | 3955 | 1274 | 942 | 783 | 1225 | 326 |

**Table S2** Pearson’s correlation coefficients (r) between environmental factors and α-diversity metrics of soil bacterial community in maize cropping systems.

| Environmental factors | OTU richness | Shannon–Wiener index |
| --- | --- | --- |
| pH | **0.409^*^** | 0.223 |
| Organic carbon | 0.044 | 0.047 |
| Total nitrogen | -0.104 | -0.063 |
| Available nitrogen | -0.103 | -0.013 |
| Available phosphorus | -0.152 | -0.155 |
| Available potassium | 0.174 | 0.196 |
| Mean annual precipitation | **-0.398^*^** | **-0.660^***^** |
| Mean annual temperature | -0.254 | **-0.449^**^** |

Data in bold indicate significant correlations, * means *P* < 0.1, ** means *P* < 0.05, *** means *P* < 0.01.

**Table S3** PERMANOVA of the environmental factors correlated with the bacterial beta-diversity for CAP analysis in maize cropping systems.

| Environmental factors | df | Sum of squares | R^2^ | Pseudo-F | P-value | Signif. |
| --- | --- | --- | --- | --- | --- | --- |
| pH | 1 | 0.0499 | 0.0929 | 2.653 | 0.044 | * |
| Mean annual precipitation | 1 | 0.1486 | 0.2768 | 7.904 | 0.002 | ** |
| Residual | 18 | 0.3384 | 0.6303 |  |  |  |

Significance: 0.01 = **; 0.05 = *

df = degrees of freedom

Permutations = 9999

Overall model significance: *Pseudo-F* = 5.2784, *P* < 0.001

**Table S4** Topological properties of co-occurrence networks of soil bacterial communities in maize cropping systems.

|  | Clustering coefficient | Average path length | Network diameter | Graph density |
| --- | --- | --- | --- | --- |
| Whole network | 0.162 | 3.237 | 4.401 | 0.0128 |
| Random network | 0.0128  (±0.00005) | 2.398  (±0.0001) | 3  (±0) | 0.0128 |

The random network is an identically sized random network generated based on the Erdös–Réyni model; the topological properties are calculated as the average value of 10,000 random Erdös–Réyni networks.

**Figure S1** Constrained analysis of principal coordinates (CAP) based on Weighted Unifrac distance and environmental factors that were significantly related to bacterial variation. Pre represents mean annual precipitation.


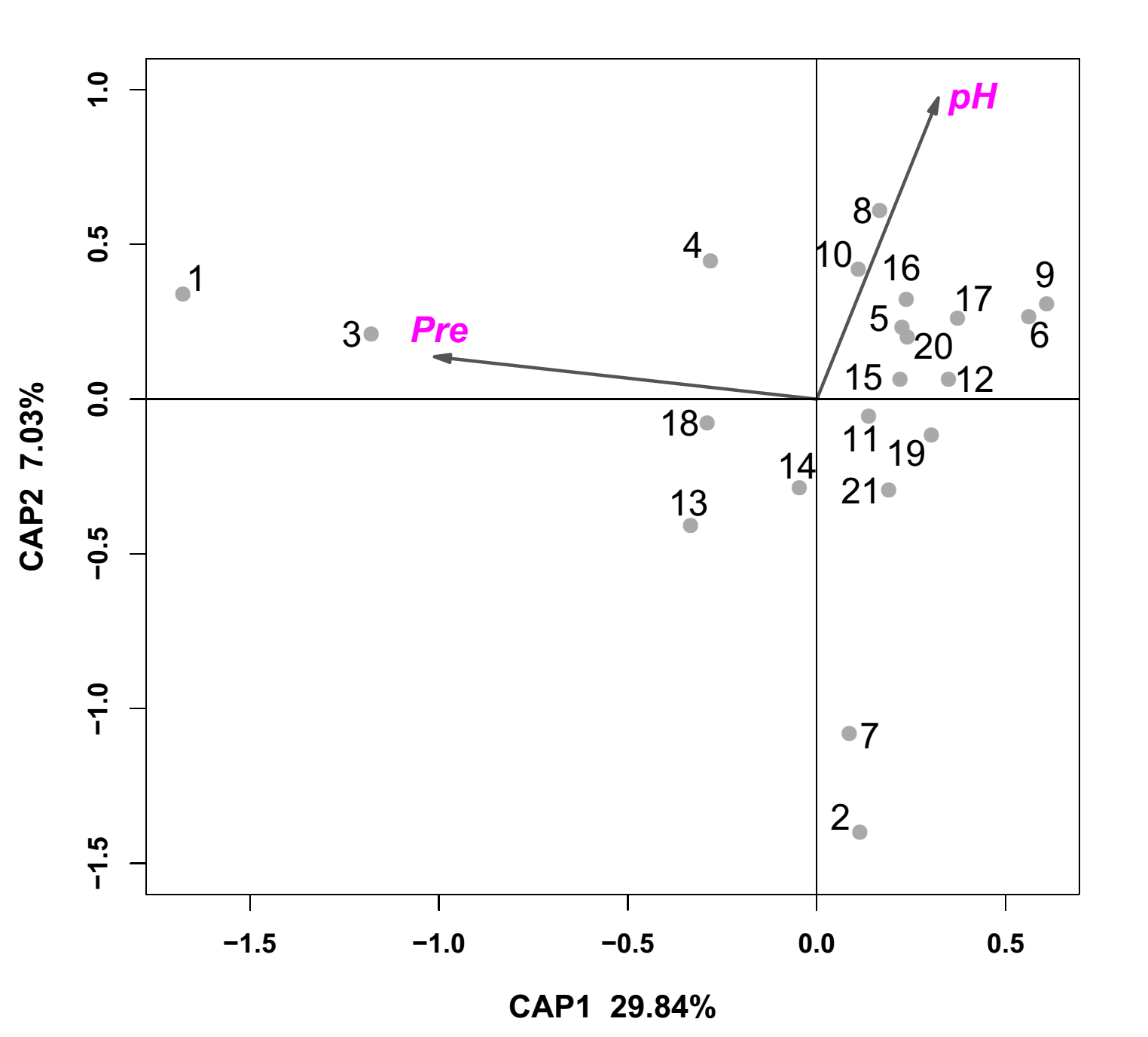


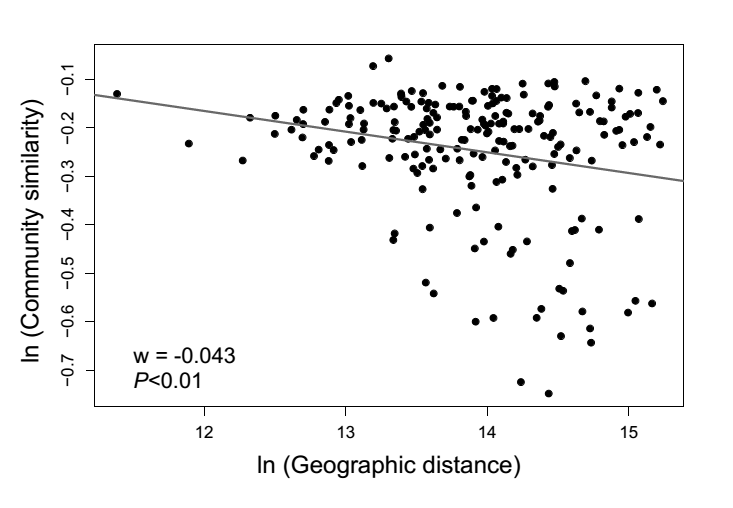
**Figure S2** Correlation between bacterial community dissimilarity (via Weighted Unifrac distance) and geographic distance in maize cropping systems. A “w” represents the slopes of the linear regression line, and a “P” represents the significance of the linear regression model.

**Figure S3** Weighted UniFrac distance principal coordinate analysis (PCoA) of bacterial communities among soil types in maize cropping systems.


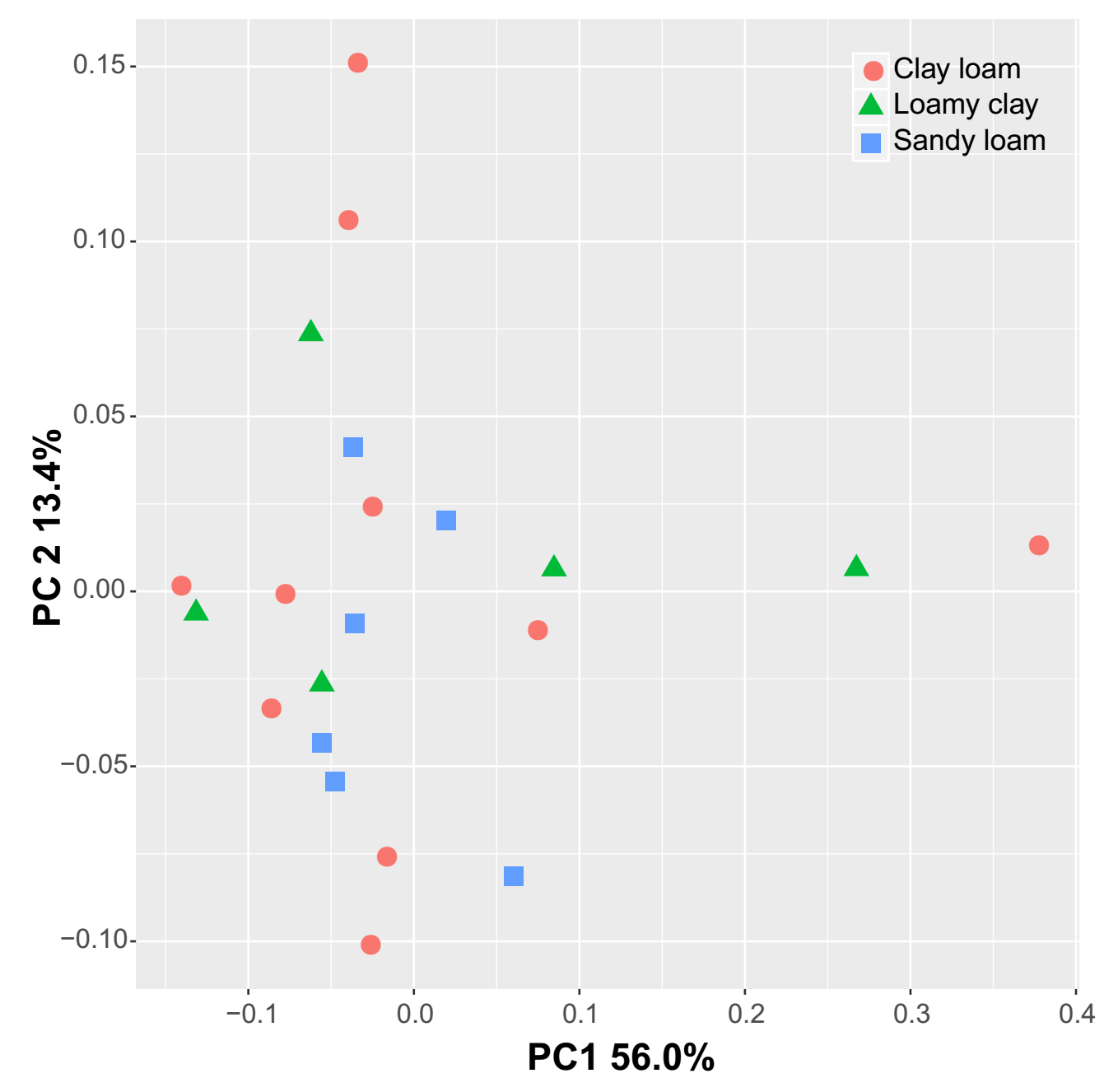


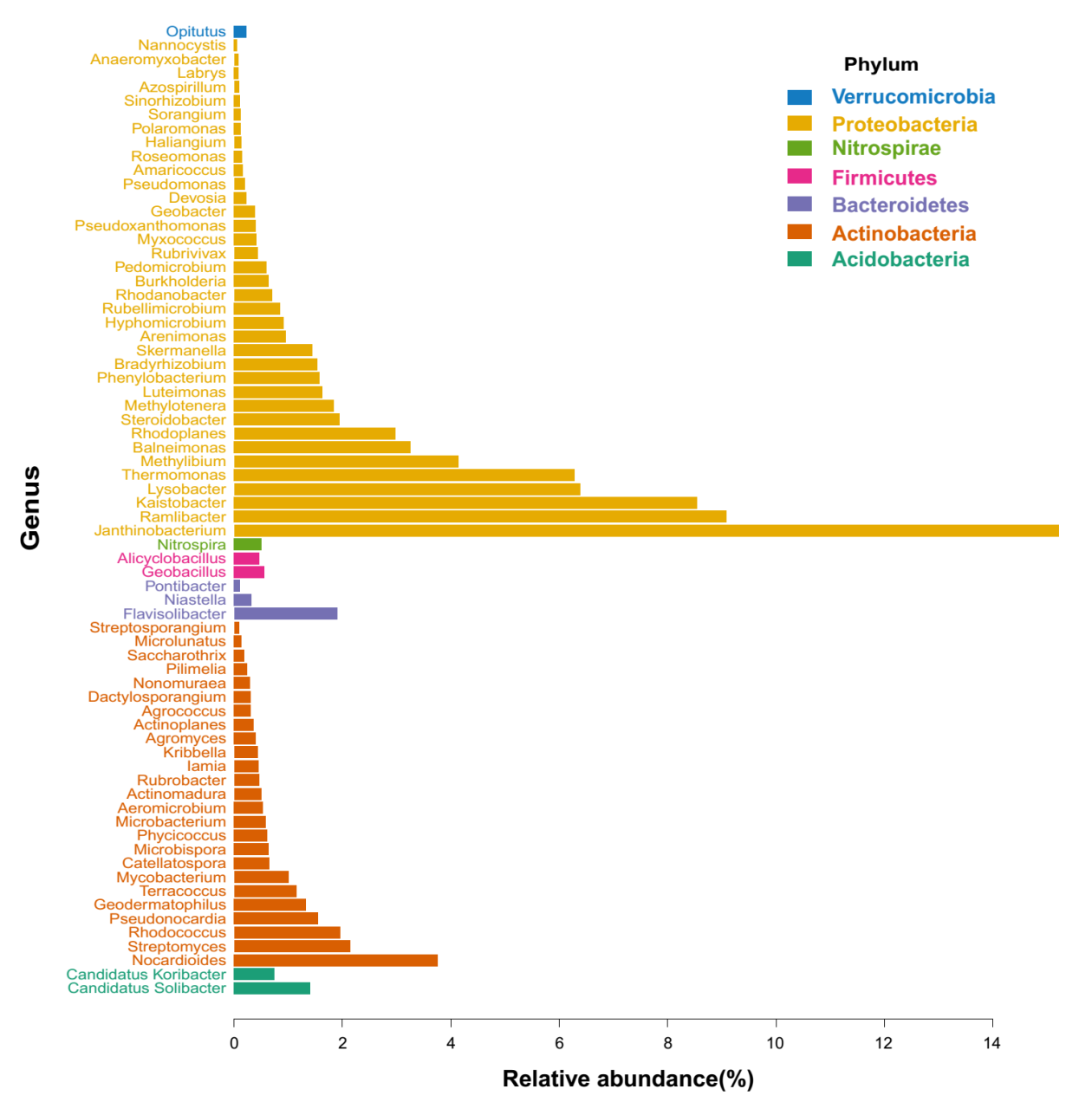
**Figure S4** The taxonomic distribution of the core microbiome at the genus level in maize cropping systems.

**Figure S5** The contributions of core microbiome to the total bacterial community dissimilarity in maize cropping systems based on Bray–Curtis distance.


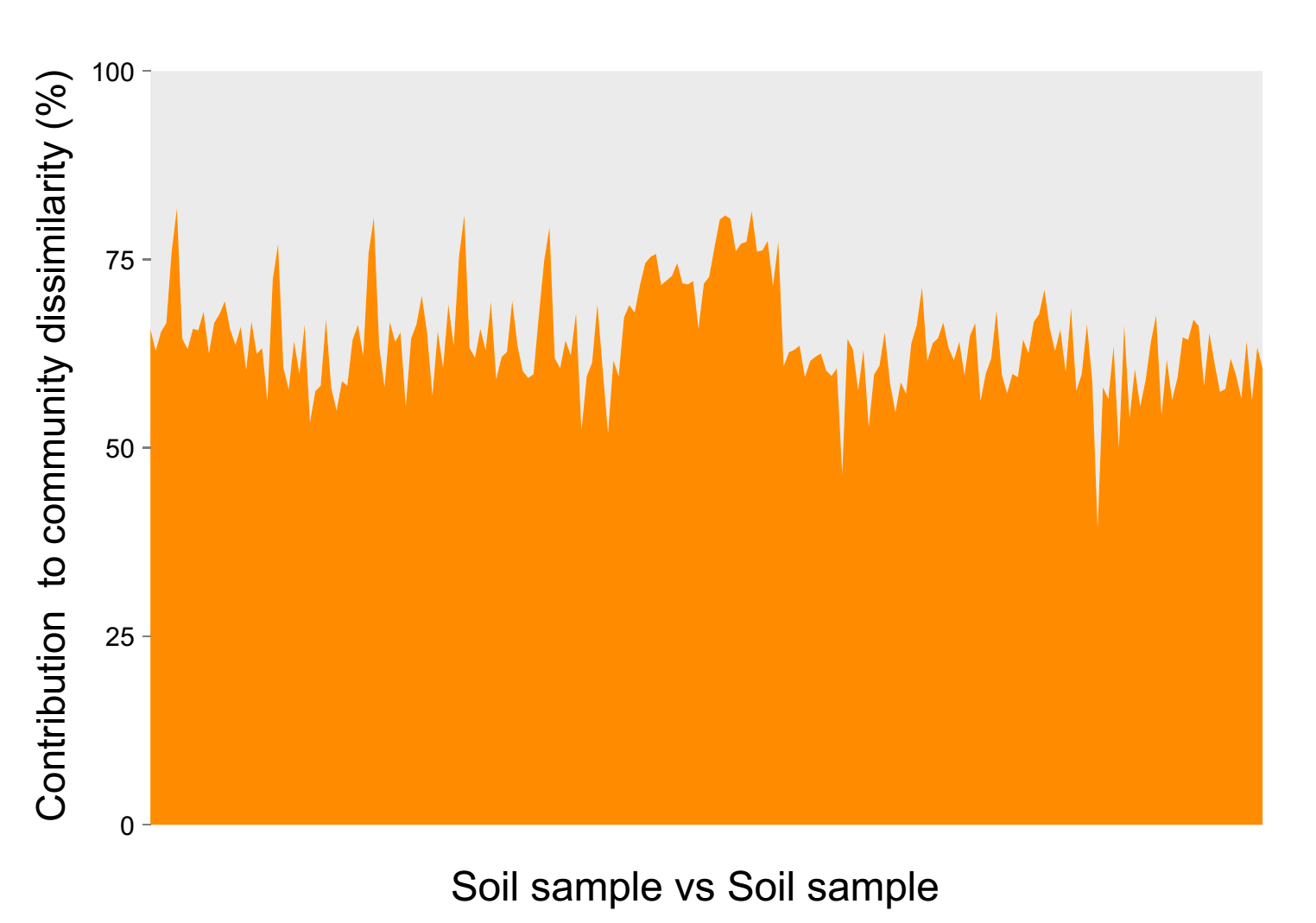


**Figure S6** Network of co-occurring bacterial genera based on correlation analysis. A connection stands for a strong (Spearman correlation coefficient, r >0.6) and significant (*P* <0.01) correlation. The size of each node is proportional to the betweenness centrality value; the thickness of the connection between two nodes (edge) is proportional to the value of the
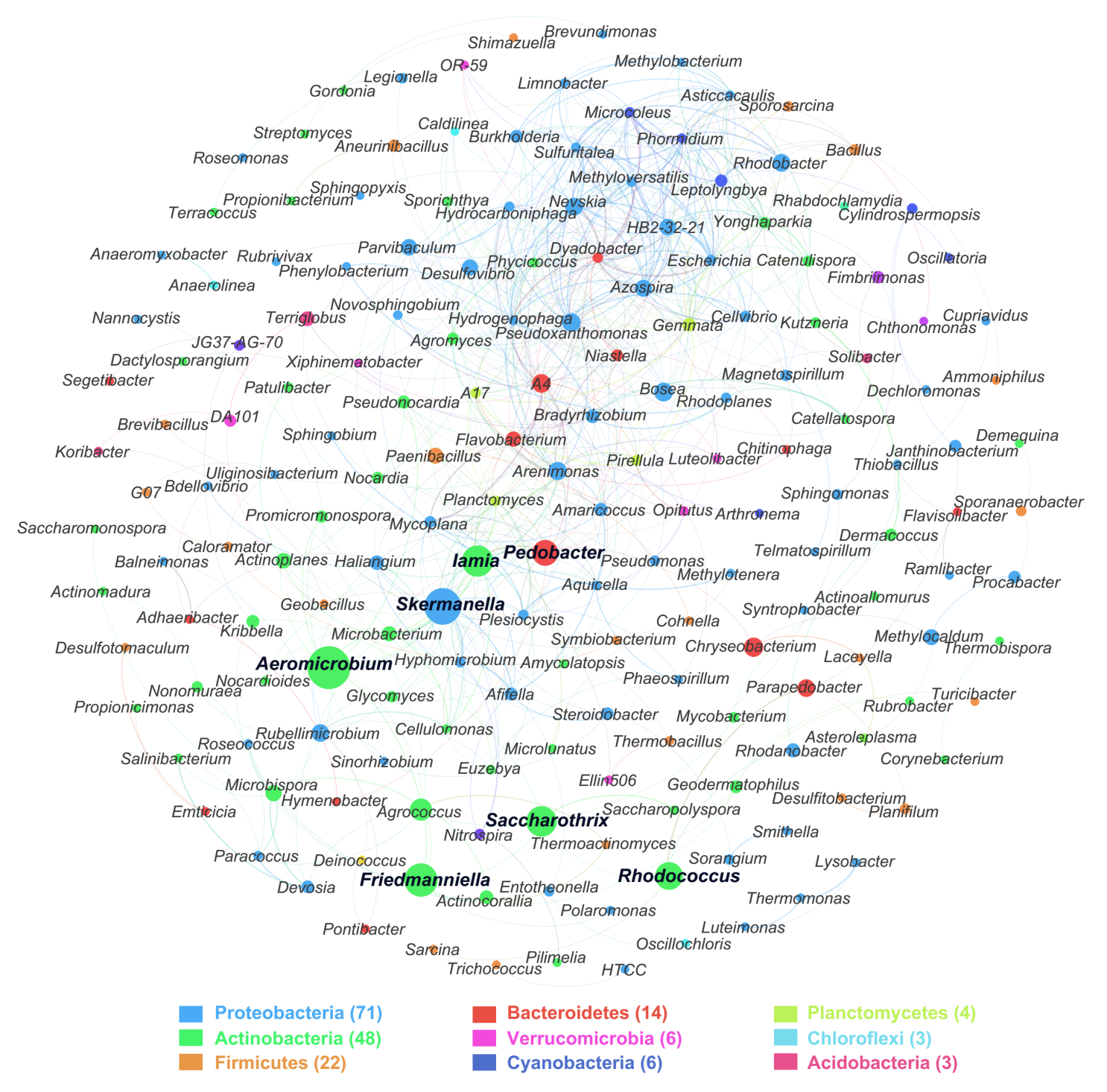
Spearman correlation coefficients. The nodes colors are based on phylum.
